# The Association between Patient Characteristics and the Efficacy and Safety of Selinexor in Diffuse Large B-Cell Lymphoma in the SADAL Study

**DOI:** 10.3390/cancers14030791

**Published:** 2022-02-04

**Authors:** Josée M. Zijlstra, George Follows, Rene-Olivier Casasnovas, Joost S. P. Vermaat, Nagesh Kalakonda, Sylvain Choquet, Brian Hill, Catherine Thieblemont, Federica Cavallo, Fatima De la Cruz, John Kuruvilla, Nada Hamad, Ulrich Jaeger, Paolo Caimi, Ronit Gurion, Krzysztof Warzocha, Sameer Bakhshi, Juan-Manuel Sancho, Michael Schuster, Miklos Egyed, Fritz Offner, Theodoros P. Vassilakopoulos, Priyanka Samal, Matthew Ku, Jenny Xu, Kelly Corona, Kamal Chamoun, Jatin Shah, Miguel Canales, Marie Maerevoet

**Affiliations:** 1 Department of Hematology, Amsterdam UMC, Cancer Center, Vrije Universiteit, De Boelelaan 1117, 1081HV Amsterdam, The Netherlands; 2 Department of Haematology, Cambridge University Hospitals NHS Foundation Trust, Cambridge CB2 0QQ, UK; george.follows@addenbrookes.nhs.uk; 3 Department of Hematology, University Hospital F. Mitterrand and INSERM 1231, 21000 Dijon, France; olivier.casasnovas@chu-dijon.fr; 4 Department of Hematology, Leiden University Medical Center, Albinesdreef 2, 2333 ZA Leiden, The Netherlands; J.S.P.Vermaat@lumc.nl; 5 Department of Molecular and Clinical Cancer Medicine, University of Liverpool, Liverpool L69 3GE, UK; NageshK@liverpool.ac.uk; 6 Hematology, Hôpital Pitié Salpêtrière, 47-83 Bd de l'Hôpital, 75013 Paris, France; sylvain.choquet@aphp.fr; 7 Department of Hematology and Medical Oncology, Taussig Cancer Institute, Cleveland Clinic, Cleveland, OH 44195, USA; HILLB2@ccf.org; 8 Hemato-Oncology, APHP, Saint-Louis Hospital & Paris University, 75010 Paris, France; catherine.thieblemont@aphp.fr; 9 Division of Hematology, Department of Molecular Biotechnologies and Health Sciences, University of Torino/AOU, Città della Salute e della Scienza di Torino, 10126 Torino, Italy; f.cavallo@unito.it; 10 Hospital Universitario Virgen del Rocio, E-41013 Sevilla, Spain; fatima.cruz.sspa@juntadeandalucia.es; 11 Division of Medical Oncology and Hematology, Princess Margaret Cancer Centre, Toronto, ON M5G 2M9, Canada; John.Kuruvilla@uhn.ca; 12 Department of Hematology, St. Vincent’s Hospital Sydney, Darlinghurst, NSW 2010, Australia; Nada.Hamad@svha.org.au; 13 St Vincent's Clinical School Sydney, University of New South Wales, Sydney, NSW 2052, Australia; 14 School of Medicine, University of Notre Dame Australia, Fremantle, WA 6160, Australia; 15 Division of Hematology and Hemostaseology, Department of Medicine I, Medical University of Vienna, Waehringer Guertel 18-20, A-1090 Vienna, Austria; Ulrich.Jaeger@meduniwien.ac.at; 16 Case Comprehensive Cancer Center, School of Medicine, Case Western Reserve University, Cleveland, OH 44106, USA; caimip@ccf.org; 17 Hematology Institute, Davidoff Center, Rabin Medical Center, Petach Tikva & Tel-Aviv University, Tel-Aviv 49100, Israel; Ronitg@clalit.org.il; 18 Department of Hematology, Instytut Hematologii I Transfuzjologii, Chocimska 5, 00-791 Warsaw, Poland; krzysztof.warzocha@fwco.org.pl; 19 Department of Medical Oncology, Dr. B. R. A. Institute Rotary Cancer Hospital, All India Institute of Medical Sciences, New Delhi 110029, India; sameerbakhshi@aiims.edu; 20 Clinical Hermatology Department, Hospital Germans Trias i Pujol, Institut Català d'Oncologia, Universitat Autònoma de Barcelona, 08916 Badalona, Spain; jsancho@iconcologia.net; 21 Stony Brook Cancer Center, Stony Brook University Hospital, Stony Brook, NY 11794, USA; Michael.Schuster@stonybrookmedicine.edu; 22 Department of Hematology, Teaching Hospital Mór Kaposi, Tallian Gy. U 20-32, H-7400 Kaposvár, Hungary; dregyedmiklos@yahoo.com; 23 Department of Hematology, Ghent University Hospital, 9000 Ghent, Belgium; Fritz.Offner@UGent.be; 24 Department of Hematology, National and Kapodistrian University of Athens, 15772 Athens, Greece; tvassilak@med.uoa.gr; 25 Hematology-Hemato-Oncology, Institute of Medical Sciences & SUM Hospital, Bhubaneswar 751003, India; priyankasamal@soa.ac.in; 26 Department of Haematology, St.Vincent’s Hospital, University of Melbourne, Melbourne, VIC 3065, Australia; matthew.ku@svha.org.au; 27 Karyopharm Therapeutics, Newton, MA 02459, USA; jxu@karyopharm.com (J.X.); kcorona@karyopharm.com (K.C.); kamal.chamoun@karyopharm.com (K.C.); jshah@karyopharm.com (J.S.); 28 Department of Hematology, Autonoma University, La Paz University Hospital, 28046 Madrid, Spain; miguel.canales@salud.madrid.org; 29 Department of Hematology, Institut Jules Bordet, 1070 Brussels, Belgium; marie.maerevoet@bordet.be

**Keywords:** selinexor, diffuse large B-cell lymphoma, exportin 1, SADAL study

## Abstract

**Simple Summary:**

Diffuse large B-cell lymphoma (DLBCL) is a complex disease. A combination of immunotherapy and chemotherapy is used to treat DLBCL at initial diagnosis. Additional treatments are available when DLBCL does not respond to initial treatment; however, for many patients, DLBCL will stop responding to treatment (relapse) or may not respond at all (refractory). Selinexor is a novel, oral medication that belongs to a class of drugs called selective inhibitors of nuclear export, and it works by killing cancer cells in patients with DLBCL that has relapsed after or is refractory to at least two treatments. When deciding on a course of treatment, it is useful for physicians to know which frequently described clinical characteristics of DLBCL affect the activity and tolerability of selinexor. We found that selinexor showed similar activity and tolerability across most of the frequently described clinical characteristics assessed.

**Abstract:**

Selinexor, an oral selective inhibitor of nuclear export, was evaluated in the Phase 2b SADAL study in patients with diffuse large B-cell lymphoma (DLBCL) who previously received two to five prior systemic regimens. In post hoc analyses, we analyzed several categories of patient characteristics (age, renal function, DLBCL subtype, absolute lymphocyte count, transplant status, number of prior lines of therapy, refractory status, Ann Arbor disease stage, and lactate dehydrogenase) at baseline, i.e., during screening procedures, to determine their potential contributions to the efficacy (overall response rate [ORR], duration of response [DOR], overall survival [OS]) and tolerability of selinexor. Across most categories of characteristics, no significant difference was observed in ORR or DOR. OS was significantly longer for patients < 65 vs. ≥ 65 years, and for those with lymphocyte counts ≥ 1000/µL vs. < 1000/µL or lactate dehydrogenase ≤ ULN vs. > ULN. The most common adverse events (AEs) across the characteristics were thrombocytopenia and nausea, and similar rates of grade 3 AEs and serious AEs were observed. With its oral administration, novel mechanism of action, and consistency in responses in heavily pretreated patients, selinexor may help to address an important unmet clinical need in the treatment of DLBCL.

## 1. Introduction

Diffuse large B-cell lymphoma (DLBCL) is a complex disease that progresses rapidly and has variable clinical outcomes. It is the most common subtype of non-Hodgkin lymphoma (NHL) accounting for 40% of NHL cases worldwide [1]. Although DLBCL may be diagnosed in people of all age groups, its incidence is highest among people 65–74 years [2].

Clinical characteristics associated with a disease may affect the selection and outcome of treatment regimens. For DLBCL, the immunochemotherapy combination of rituximab with cyclophosphamide, doxorubicin, vincristine, and prednisone is the established frontline treatment, curing up to 60% of patients treated with a full course of therapy [3,4,5]. Potentially curative salvage therapy for patients with relapsing or refractory (R/R) disease includes high-dose chemotherapy followed by autologous stem cell transplantation (ASCT). Chimeric antigen receptor (CAR)-T cell therapy is a more recently approved treatment for R/R DLBCL and, similar to ASCT, CAR-T is limited by availability and patient eligibility [6,7]. Patients who are not candidates for intensive chemotherapy, ASCT, or CAR-T therapy are offered non-curative therapies including chemotherapy combinations with targeted agents or targeted agents alone.

Exportin 1 (XPO1) is the major nuclear exporter of tumor suppressor proteins (TSPs) such as p53, FOXO, IκB, and Rb, and the mRNA cap-binding protein eIF4E [8,9,10]. XPO1 overexpression is observed in many types of cancer including DLBCL, and higher levels of XPO1 are associated with poor prognosis in DLBCL [11] and other cancers [12]. XPO1 overexpression leads to the nuclear export and functional inactivation of TSPs and enhances the levels of eIF4E-associated oncoproteins such as c-Myc [13,14,15]. Selinexor is a first-in-class selective inhibitor of nuclear export (SINE) compound that selectively binds and inactivates XPO1. Inactivation of XPO1 forces the nuclear retention and reactivation of cell cycle regulators and reduces the concentration of the oncoproteins, several of which play critical roles in NHL [8,16,17,18].

Based on results from the Selinexor Against Diffuse Aggressive Lymphoma (SADAL) study [19], the US Food and Drug Administration in 2020 approved the use of single-agent selinexor for the treatment of adult patients with DLBCL that is de novo or transformed from follicular lymphoma after at least two prior lines of systemic therapy [20].

In this report, we describe the results of the post hoc analyses of baseline characteristics for patients with R/R DLBCL from the SADAL study. The objective of the analyses in this report was to determine whether clinically important differences exist in the efficacy and safety of selinexor based on clinical characteristics frequently described in patients with DLBCL.

## 2. Materials and Methods

### 2.1. Study Design

SADAL was an open-label, phase 2b study carried out at 59 sites in 19 countries. Details on the design of the study (NCT02227251) have been reported elsewhere [19].

### 2.2. Endpoints

The efficacy endpoints examined in these post hoc analyses include overall response rate (ORR), the primary endpoint of the SADAL study, duration of response (DOR), and overall survival (OS).

### 2.3. Baseline Characteristics

In these post hoc analyses, we examined the clinical characteristics recorded at baseline, i.e., during screening procedures for patients enrolled in the SADAL study, to determine whether the characteristics, which are frequently described in patients with DLBCL, are associated with differences in the efficacy and safety of selinexor. The clinical characteristics included in these analyses are: (1) age, <65 years vs. ≥65 years; (2) renal function, creatinine clearance (CrCL) ≤ 60 mL/min vs. > 60 mL/min; (3) DLBCL subtypes, germinal center B-cell (GCB) vs. non-GCB; (4) absolute lymphocyte count (ALC), <1000/µL vs. ≥1000/µL; (5) prior ASCT or transplant ineligible; (6) number of prior lines of therapy, 2 vs. ≥3 prior lines; (7) refractory disease status, progressive disease (PD) < 6 months from first-line therapy (primary refractory) vs. PD ≥ 6 months from first-line (non-primary refractory); (8) Ann Arbor stage 1 or 2 vs. stage 3 or 4; and (9) lactate dehydrogenase (LDH) level, >ULN vs. ≤ULN.

### 2.4. Statistical Analyses

The primary analysis of the SADAL study was based on a one-sided exact test at an α level of 0·025 to detect a minimum of 25% of patients with a partial response or better against a value of 15% under the null hypothesis with 80% power [19]. These post hoc analyses of the SADAL study included the modified intention-to-treat (mITT) population, i.e., 134 patients who received 60 mg selinexor twice weekly until disease progression or unacceptable toxicity.

At data cutoff (1 August 2019), the primary analysis of ORR was calculated in the mITT population with the exact 2-sided 95% CI [19]. Summary statistics were computed and displayed for each subgroup and according to each assessment timepoint.

Summary statistics for continuous variables minimally included number, mean, standard deviation, minimum, median, and maximum. Frequencies and percentages are presented for categorical variables and a 2-sided 95% exact confidence interval (CI) for ORR. The chi-squared test was used to compare proportions between subgroups. For time-to-event variables, the Kaplan–Meier method was used for descriptive summaries. Log-rank test and Cox proportional hazards model were used to compare survival distributions between subgroups. Statistical analyses were performed using SAS (version 9.4).

## 3. Results

The SADAL study was initially designed as a randomized trial to evaluate two doses of selinexor, 60 mg and 100 mg, administered twice weekly. A preplanned interim analysis showed that the higher dose of 100 mg had similar levels of efficacy and was associated with greater toxicity compared with the 60-mg dose; consequently, the 100 mg dose was discontinued [19]. The median time from last systemic therapy to the start of treatment with selinexor was 5.4 months.

### 3.1. Demographics

The demographics of the patients included in this analysis are summarized in Table 1. The median age was 67 years with 44.8% of patients ≥70 years. Most patients were men (59%), median of two prior treatment regimens (range 2–5), 41% of patients received three or more prior treatment regimens, and 29.9% previously underwent an ASCT.

### 3.2. Duration of Selinexor Treatment

The median duration of selinexor treatment was 9 weeks (range 1–193) for the 134 patients in these post hoc analyses who comprised the mITT population in the SADAL study. Patients with a longer median duration of treatment with selinexor were those who were <65 years (13.5 weeks), prior ASCT (16 weeks), or with LDH ≤ ULN (15 weeks).

The median time on selinexor for responders was 214 days (range 53–1351) compared with 43 days (range 1–288) for those who did not have at least a PR on treatment. Among the patients who received at least two cycles of treatment, ORR was 52%.

### 3.3. Efficacy

**Figure 1:**
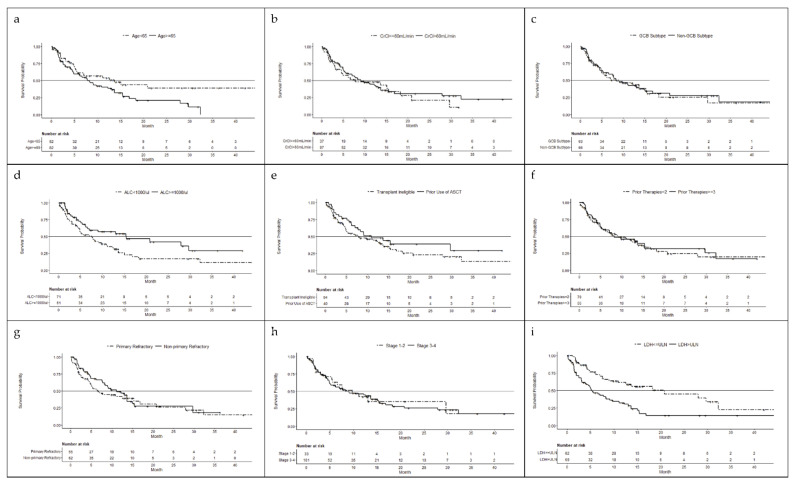
Kaplan–Meier estimated overall survival based on baseline characteristics: (**a**) age, <65 years vs. ≥65 years; (**b**) renal function, creatinine clearance (CrCL) ≤ 60 mL/min vs. >60 mL/min; (**c**) DLBCL subtypes, germinal center (GCB) vs. non-GCB; (**d**) absolute lymphocyte count (ALC), <1000/µL vs. ≥1000/µL; (e) prior ASCT or transplant ineligible; (**f**) number of prior lines of therapy, 2 vs. ≥3 prior lines; (**g**) refractory disease status, progressive disease (PD) < 6 months from first line therapy (primary refractory) vs. PD ≥ 6 months from first line (non-primary refractory); (**h**) Ann Arbor stage 1 or 2 vs. stage 3 or 4; (**i**) lactate dehydrogenase (LDH) level, >ULN vs. ≤ULN. Abbreviations: ALC = absolute lymphocyte count, ASCT = autologous stem cell transplant, CrCl = creatinine clearance, DLBCL = diffuse large B-cell lymphoma, GCB = germinal center B-cell, LDH = lactate dehydrogenase, ULN = upper limit of normal.

The relationship between the baseline characteristics and the efficacy endpoints of selinexor are summarized in Table 2. Kaplan–Meier analyses of OS based on each characteristic are shown in Figure 1.

#### 3.3.1. Age

ORR was numerically higher in patients <65 years (36.5%) compared with patients ≥65 years (24.4%) (*p* = 0.19); however, DOR was similar (9.2 months vs. 9.7 months, hazard ratio [HR] 0.95 [95% CI 0.37–2.48], *p* = 0.94) between the age groups at baseline. Median OS was 7.8 months for older patients compared with the significantly longer median OS of 13.7 months for patients <65 years (HR 1.65 [95% CI 1.03–2.64], *p* = 0.04).

#### 3.3.2. Renal Function

ORR was similar for patients with baseline CrCl ≤ 60 mL/min or >60 mL/min (29.7% vs. 28.9% [*p* = 1.00]), and DORs were numerically though not significantly different for patients with CrCl ≤60 mL/min vs. >60 mL/min (23.0 months vs. 9.2 months, HR 0.51 [95%CI 0.16–1.60], *p* = 0.24). Median OS for patients with CrCl ≤60 mL/min was not significantly different than that for patients with CrCl > 60 mL/min (7.8 months vs. 9.1 months, HR 1.14 [95% CI 0.70–1.42], *p* = 0.59).

#### 3.3.3. Germinal Center B-Cell versus Non-Germinal Center B-cell

ORR for patients with GCB was numerically (31.7%) but not significantly higher than for non-GCB (24.2%) DLBCL (*p* = 0.45) and median DOR was 23 months and 9.3 months, respectively (HR 1.58 [95% CI 0.55–4.53], *p* = 0.39). OS was similar with 9.0 months for patients with the GCB subtype and 8.3 months for patients with non-GCB (HR 0.95 [95% CI 0.61–1.50], *p* = 0.84).

#### 3.3.4. Absolute Lymphocyte Count

There was no statistically significant difference in ORR between patients with baseline ALC < 1000/µL or ≥1000/µL, with ORR of 25.4% vs. 32.8% (*p* = 0.45). A trend toward higher DOR was observed in patients with ALC ≥ 1000/µL (4.9 months vs. 23 months, HR 1.83 [95%CI 0.68–4.97], *p* = 0.23) while median OS was significantly shorter for patients with ALC < 1000/µL (7.6 months vs. 15.5 months, HR 1.79 [95% CI 1.12–2.84], *p* = 0.01) as previously reported in DLBCL [21].

#### 3.3.5. Prior ASCT vs. Transplant Ineligible

As compared with patients who were transplant ineligible, patients who received a prior ASCT had a significantly better ORR (42.5% vs. 23.4% [*p* = 0.04]) while median DOR was similar (8.4 months vs. 9.7 months [*p* = 0.93]). Median OS for patients with prior ASCT was 10.9 months versus 7.8 months for those who were transplant ineligible (HR 0.72; 95% CI 0.44–1.17; *p* = 0.18). The reasons that patients were ineligible for ASCT may have included one or more of the following: persistent disease (*n* = 30), failure to collect stem cells (*n* = 5), age (*n* = 46), frailty (*n* = 13), inadequate performance status (*n* = 10), renal or hepatic dysfunction (*n* = 3), comorbidities (n = 7), cardiac dysfunction (*n* = 9), pulmonary dysfunction (*n* = 3), infection risk (*n* = 2), patient’s refusal (*n* = 6), or financial reasons (*n* = 6).

#### 3.3.6. Prior Therapy

ORR for patients who previously received 2 lines of therapy was 27.8% versus 30.9% for those with ≥3 lines (*p* = 0.85); median DORs were 10.4 months and 8.4 months (HR 1.51 [95% CI 0.58–3.94], *p* = 0.40), respectively. Median OS for patients with 2 prior lines of therapy was 9.1 months versus 8.2 months for those with ≥3 lines (HR 0.93; 95% CI 0.60–1.46; *p* = 0.76).

#### 3.3.7. Disease Refractory Status

The ORR in patients with DLBCL that progressed within 6 months of first-line therapy (defined as primary refractory disease) was numerically lower but not significantly different from that for patients with PD ≥ 6 months after first-line therapy (21.8% vs. 37.1%, *p* = 0.11). Median DORs were 9.7 months and 9.3 months (HR 0.84 [95% CI 0.28–2.54], *p* = 0.76), respectively. Median OS for patients with primary refractory disease was 6.6 months versus 11.1 months for those with disease that progressed ≥6 months after first-line therapy (HR 0.84; 95% CI 0.52–1.35; *p* = 0.46).

#### 3.3.8. Ann Arbor Stage 1 or 2 vs. 3 or 4

Patients with disease stage 1 or 2 versus 3 or 4 at screening had similar ORRs (30.3% vs. 28.7% [*p* = 1.00], respectively). Median DOR was not reached for patients with stage 1 or 2 disease and was 4.9 months for those with stage 3 or 4 disease. Median OS was similar with 9.8 months for patients with stage 1 or 2 disease and 9.0 months for patients with stage 3 or 4 (HR 0.97 [95% CI 0.57–1.64], *p* = 0.91). ORR and OS were not statistically different for patients with extranodal disease versus those without it: ORRs were 29.5% vs. 28.2% (*p* = 1.00), and OS were 8.2 months vs. 11.2 months (HR 1.03 [95% CI 0.62–1.72], *p* = 0.91).

#### 3.3.9. Lactate Dehydrogenase >ULN vs. ≤ULN

Of the patients with baseline LDH > ULN, 17.4% had a response to treatment while 41.9% of those with baseline LDH ≤ ULN had an ORR (*p* = 0.004); median DOR was 9.7 months for patients with LDH > ULN and 10.4 months for patients with LDH ≤ ULN. Median OS was significantly shorter for patients with LDH > ULN (5.4 months vs. 20.8 months, HR 2.33 [95% CI 1.45–3.72], *p* = 0.0003).

Multivariate analysis including age, ALC, and LDH showed that only LDH ≤ ULN was independently associated with higher OS (HR = 2.35 [95% CI 1.45–3.79]) (see Table 2).

### 3.4. Safety

The safety profile of these post hoc analyses is based on the baseline characteristics and adverse events (AEs) reported by patients during the SADAL study.

Overall, 132 of the 134 patients (98.5%) included in this analysis experienced at least one AE during the study. Across the baseline characteristics, thrombocytopenia (rarely with clinically significant bleeding) and nausea occurred in at least 50% of the patients (Table 3). There were no notable differences in AEs between the categories for each characteristic.

The frequency of grade 3 or higher AEs ranged from 82% to 90% for the categories of each characteristic and was similar for most characteristics including age (<65 years, 82.7%; ≥65 years, 85.4%), DLBCL subtypes (GCB, 81.0%; non-GCB, 86.4%), ALC (<1000/µL, 85.9%; ≥1000/µL 82.0%), disease stage (1 or 2, 81.8%; 3 or 4, 85.1%), and LDH (>ULN, 87.0%; ≤ULN, 82.3%), prior therapies (<2 therapies, 84.8%; ≥3 therapies, 83.6%), and refractory disease (PD < 6 months after first-line therapy, 83.6%; PD ≥ 6 months, 85.5%). Patients with CrCl ≤ 60 mL/min had a slightly higher incidence of grade 3 or higher AEs than patients with CrCl > 60 mL/min (89.2% vs. 82.5%). The incidence of grade 3 or higher AEs was higher for patients with prior ASCT versus transplant ineligible (90.0% vs. 81.9%).

Serious AEs (SAEs) occurred at a similar frequency for most characteristics including age (<65 years, 48.1%; ≥65 years, 46.3%), DLBCL subtype (GCB, 44.4%; non-GCB, 48.5%), ALC (<1000/µL, 49.3%; ≥1000/µL 44.3%), transplant status (prior ASCT, 47.5%; transplant ineligible, 46.8%), number of prior lines of therapy (2 prior lines, 48.1%; ≥3 prior lines, 45.5%), refractory status (PD <6 months after first-line therapy, 45.5%; PD ≥6 months, 53.2%), and LDH (>ULN, 56.5%; ≤ULN, 37.1%). Patients with CrCl >60 mL/min had a slightly higher frequency of SAEs (49.5%) than patients with CrCl of ≤60 mL/min (40.5%). The incidence of SAEs was notably higher for patients with stage 3 or 4 disease (53.5%) compared with those with stage 1 or 2 disease (27.3%) (*p* = 0.02), consistent with a significant contribution of disease extent to the development of an SAE.

Across baseline characteristics, 17.2% of patients with at least one AE withdrew from treatment with selinexor. Of all patients in this analysis, 48.5% had a dose reduction and a majority (64.2%) had at least one dose that was interrupted or withheld (Table 4). In the SADAL study, the most common dose reduction AEs (3 or more patients [≥2%]) were thrombocytopenia in 30 patients (23.6%), neutropenia in 11 (8.7%), fatigue in 6 (4.7%), and nausea in 4 (3.1%). Common reasons for dose interruption of more than 2 weeks included thrombocytopenia in 18 (14.2%) patients, fatigue in 3 (2.4%), and asthenia in 3 (2.4%).

Of the 134 patients in the mITT, 27 patients (20.1%) died within 30 days of receiving the last dose of selinexor: 22 (16.4%) died due to progressive disease and 5 patients (3.7%) died of an unrelated AE.

## 4. Discussion

Clinical characteristics associated with DLBCL contribute to outcomes for patients receiving treatment for the disease. Selinexor is a novel, oral nuclear export inhibitor; it is important to determine which clinical parameters of DLBCL are associated with the drug’s activity and tolerability. In these post hoc analyses, we examined multiple frequently described baseline clinical characteristics of patients with R/R DLBCL who were enrolled in the SADAL study. The baseline clinical characteristics examined in these analyses included age, renal function (CrCl), DLBCL subtype, ALC, prior ASCT, number of prior lines of therapy, refractory status, Ann Arbor stage, and LDH level.

It has already been established that age is not a factor in the metabolism of selinexor and has no clinically significant effect on the pharmacokinetics of the drug [22]. In this analysis, we showed that patients with R/R DLBCL who were ≥65 years had a similar clinical benefit when compared with those <65 years and treated with selinexor with comparable ORR, DOR, and perhaps most importantly, overall tolerability. As expected, younger patients (<65 years) had significantly longer OS (*p* = 0.04) than those ≥65 years, most likely due to comorbid medical conditions in the older population [23]; AE rates were not significantly different in these two populations (Table 3). Doses of treatment were interrupted or withheld for a small proportion of patients <65 years compared with patients ≥65 years (59.6% vs. 67.1%). These results indicate that single-agent oral selinexor can induce durable responses with similar tolerability in younger and older patients with heavily pretreated DLBCL. These observations are particularly important for older patients who may prefer a non-parenteral agent that can be taken at home with proper monitoring.

In addition to age, selinexor metabolism is not affected by renal function with no clinically significant effect on the pharmacokinetics of selinexor [22]. Additionally, renal clearance is a minor route for the elimination of selinexor with most excreted in feces by the hepatobiliary route as unchanged drug or metabolites (unpublished data). In this analysis, patients with reduced renal function (CrCl ≤ 60 mL/min) and those with normal function (CrCl > 60 mL/min) had similar outcomes when treated with selinexor 60 mg twice weekly, unlike other settings in which patients with newly diagnosed DLBCL and lower renal function were associated with lower overall survival [24]. The safety profile in the current analysis was similar between the categories in the proportion of patients who experienced AEs, the types of AEs, and deaths within 30 days of the last dose. This safety profile is similar to that from previous assessments in which patients with multiple myeloma and moderate (CrCl 30–60 mL/min) or severe (CrCl < 30 mL/min) renal impairment had a profile similar to that of selinexor in patients with normal renal function or mild renal impairment (unpublished data), which suggests that treatment with selinexor does not require dose adjustments in patients with renal dysfunction and R/R DLBCL.

Patients who were previously treated for DLBCL had strong and durable responses when treated with single-agent selinexor, regardless of GCB or non-GCB subtype [25].

Low ALC is also a known poor prognostic marker in patients with DLBCL [21,26,27,28]. For patients treated in the SADAL trial, the ORR was similar between patients regardless of baseline ALC; however, significantly longer OS (*p* = 0.01) was observed in patients with baseline ALC ≥ 1000 µL; responses in these patients tended to last longer. These results are consistent with reports in the literature regarding the poor prognosis of baseline lymphocyte count < 1000/µL, but suggest that the anti-lymphoma activity of selinexor is minimally affected by baseline ALC.

Patients with prior ASCT, compared with those who were transplant ineligible, had a significantly higher ORR; DOR was similar with a trend for a longer OS. These results are not unexpected since patients with prior ASCT were generally fitter and had substantial responses to second-line therapy which permitted the transplant.

Numerous therapies are available to treat R/R DLBCL; however, there is no standard of care after three or more lines. When treated with selinexor, ORR, DOR, and OS were comparable for patients who had 2 versus ≥3 lines of previous treatment, consistent with the novel, non-cross resistant mechanism of action for selinexor. Of note, the population enrolled in the SADAL study represented patients with aggressive disease as reflected in the median time of 5.4 months since last treatment to initiation of selinexor compared with the L-MIND study in which the median time was 9 months for patients treated with tafasitamab plus lenalidomide [29]. Additionally, the analyses in this report showed that ORR was significantly higher for patients who had undergone ASCT than for those without it. ORR was also higher for patients with a response of PR or CR to the last line of therapy than for those without a response. Furthermore, two of the thirty-seven patients who did not have a response of PR or CR with any previous therapy had a response to selinexor (two PR, four CR). In patients with primary refractory DLBCL, ORR was not significantly lower than ORR for patients with non-primary refractory disease, again consistent with selinexor’s novel mechanism of action. These results are in contrast to those from the SCHOLAR-1 analysis, a large patient-level pooled retrospective analysis in refractory disease, in which outcomes were poor for most patients (73%) who did not respond to salvage therapy or were unable to receive ASCT [30]. Because the SCHOLAR-1 analysis was carried out by others, we do not have the data or information on the baseline characteristics needed to compare individual characteristics with those from the SADAL study. However, overall, the outcomes from the SADAL study were better than those published from the SCHOLAR-1 analysis.

These post hoc analyses were limited by their retrospective nature and by the small number of patients in the subgroups. For these reasons some of the comparisons did not have the power to conclude whether differences were statistically significant.

Disease stage and LDH are strong prognostic factors that are part of the IPI and R-IPI prognostic scoring system for newly diagnosed patients. However, the significance of these prognostic scores is still not validated in the relapsed/refractory setting. In the current analysis, we found that, although ORR and OS were statistically similar between patients with different disease stages, ORR was significantly higher for patients with LDH ≤ ULN and OS was significantly longer. These differences should be verified in larger studies, but strongly suggest that single-agent oral selinexor is substantially more active in patients with LDH ≤ ULN and, as a single oral therapy, may be a particularly attractive option for these patients.

## 5. Conclusions

Patients with R/R DLBCL tend to be clinically complex because of their advanced age and medical history which may include prior treatments, use of concomitant medications, comorbidities, and other medical concerns. As a result, these patients usually are unable to tolerate multiple cycles of standard combination therapies for DLBCL creating an unmet medical need, especially for patients with R/R DLBCL previously treated with multiple lines of therapy. Selinexor showed similar activity and tolerability across most of the frequently described clinical characteristics assessed here (age, renal function, DLBCL subtype, lymphocyte counts, prior ASCT, number of prior lines of therapy, refractory status), but appeared to be less active in patients with LDH levels >ULN. Notably, SAE rates were about twice as high in patients with stage 3/4 disease as compared with stage 1/2 DLBCL, consistent with a significant contribution to AEs from the tumor itself. Selinexor, with its novel mechanism of action, ease of oral administration, and ability to produce rapid and durable responses in patients with heavily pretreated disease, may help to fill this important unmet clinical need. Combination therapy studies with selinexor (NCT04442022, NCT04607772) are ongoing to determine optimal dosing and response rates/durability; these regimens are highly likely to be substantially more active than single-agent selinexor.

## Figures and Tables

**Table 1:** Demographics.

Characteristic	Age		CrCl		ALC ^a^		Transplant		Stage of Disease		LDH ^b^		Total
	<65 years (*n* = 52)	≥65 years (*n* = 82)	≤60 mL/min (*n* = 37)	>60 mL/min (*n* = 97)	<1000/µL (*n* = 71)	≥1000/µL (*n* = 61)	Prior ASCT (*n* = 40)	Transplant Ineligible (*n* = 94)	1 or 2 (*n* = 33)	3 or 4 (*n* = 101)	>ULN (*n* = 69)	≤ULN (*n* = 62)	(N = 134)
Median age, years (min, max)	57 (35, 64)	73 (65, 91)	74 (52, 91)	65 (35, 83)	67 (35, 91)	67 (44, 87)	64 (41, 77)	69.5 (35, 91)	70 (35, 87)	67 (41, 91)	65 (35, 86)	69 (41, 91)	67 (35, 91)
≥70, *n* (%)	0	60 (73.2)	26 (70.3)	34 (35.1)	33 (46.5)	26 (42.6)	13 (32.5)	47 (50.0)	18 (54.5)	42 (41.6)	30 (43.5)	30 (48.4)	60 (44.8)
Male, *n* (%)	32 (61.5)	47 (57.3)	14 (37.8)	65 (67.0)	43 (60.6)	35 (57.4)	27 (67.5)	52 (55.3)	15 (45.5)	64 (63.4)	41 (59.4)	37 (59.7)	79 (59.0)
DLBCL subtype, *n* (%)													
GCB	28 (53.8)	35 (42.7)	15 (40.5)	48 (49.5)	31 (43.7)	31 (50.8)	25 (62.5)	38 (40.4)	15 (45.5)	48 (47.5)	33 (47.8)	28 (45.2)	63 (47.0)
Non-GCB	21 (40.4)	45 (54.9)	21 (56.8)	45 (46.4)	39 (54.9)	26 (42.6)	13 (32.5)	53 (56.4)	17 (51.5)	49 (48.5)	35 (50.7)	30 (48.4)	66 (49.3)
Non-classified	3 (5.8)	2 (2.4)	1 (2.7)	4 (4.1)	1 (1.4)	4 (6.6)	2 (5.0)	3 (3.2)	1 (3.0)	4 (4.0)	1 (1.4)	4 (6.5)	5 (3.7)
No. prior regimens													
Median (min, max)	2 (2, 5)	2 (2, 5)	2 (2, 5)	2 (2, 5)	2 (2, 5)	2 (2, 5)	2 (2, 5)	2 (2, 5)	2 (2, 5)	2 (2, 5)	2 (2, 5)	2 (2, 5)	2 (2, 5)
2, *n* (%)	30 (57.7)	49 (59.8)	21 (56.8)	58 (59.8)	44 (62.0)	34 (55.7)	21 (52.5)	58 (61.7)	19 (57.6)	60 (59.4)	40 (58.0)	37 (59.7)	79 (59.0)
3, *n* (%)	13 (25.0)	20 (24.4)	12 (32.4)	21 (21.6)	15 (21.1)	17 (27.9)	14 (35.0)	19 (20.2)	8 (24.2)	25 (24.8)	19 (27.5)	13 (21.0)	33 (24.6)
4, *n* (%)	6 (11.5)	10 (12.2)	3 (8.1)	13 (13.4)	9 (12.7)	7 (11.5)	3 (7.5)	13 (13.8)	4 (12.1)	12 (11.9)	7 (10.1)	9 (14.5)	16 (11.9)
5, *n* (%)	3 (5.8)	3 (3.7)	1 (2.7)	5 (5.2)	3 (4.2)	3 (4.9)	2 (5.0)	4 (4.3)	2 (6.1)	4 (4.0)	3 (4.3)	3 (4.8)	6 (4.5)
Prior ASCT, *n* (%)	32 (61.5)	31 (37.8)	13 (35.1)	50 (51.5)	24 (33.8)	37 (60.7)	40 (100)	0	9 (27.3)	31 (30.7)	17 (24.6)	23 (37.1)	40 (29.9)
Refractory Status ^c^													
Primary, *n* (%)	22 (42.3)	33 (40.2)	15 (40.5)	40 (41.2)	27 (38.0)	28 (45.9)	11 (27.5)	44 (46.8)	11 (33.3)	44 (43.6)	27 (43.6)	26 (37.7)	55 (41.0)
Non-primary refractory, *n* (%)	23 (44.2)	39 (47.6)	16 (43.2)	46 (47.4)	34 (47.9)	26 (42.6)	23 (57.5)	39 (41.5)	17 (51.5)	45 (44.6)	26 (41.9)	36 (52.2)	62 (46.3)

Abbreviations: ALC = absolute lymphocyte count, ASCT = autologous stem cell transplant, CrCl = creatinine clearance, DLBCL = diffuse large B-cell lymphoma, GCB = germinal center B-cell, LDH = lactate dehydrogenase, max = maximum, min = minimum, ULN = upper limit of normal. ^a^ Data missing for 2 patients. ^b^ Data missing for 3 patients. ^c^ Primary refractory is defined as disease progression within 6 months of first-line therapy. Non-primary refractory disease is defined as disease progression ≥6 months after first-line therapy.

**Table 2:** Efficacy of selinexor based on baseline characteristics in the mITT population.

		ORR ^a^		DOR ^b^		PFS		OS ^b^		
Variable	No. Patients	*n* (%)	*p*-Value	Median Months	*p*-Value	Median Months	*p*-Value	Median Months	*p*-Value	Multivariate Analysis *p*-Value/HR (95%CI)
Overall	134	39 (29.1)	--	9.3	--	2.6		9.0		
Age									--	
<65 years	52	19 (36.5)	0.19	9.7	0.94	3.6	0.91	13.7	0.04	0.03/1.7 (1.05,2.78)
≥65 years	82	20 (24.4)		9.2		2.3		7.8		
CrCl										
≤60 mL/min	37	11 (29.7)	1.00	23.0	0.24	3.5	0.66	7.8	0.59	
>60 mL/min	97	28 (28.9)		9.2		2.3		9.1		
DLBCL Subtype										
GCB	63	20 (31.7)	0.45	23	0.39	3.6	0.105	9.0	0.836	
Non-GCB	66	16 (24.2)		9.3		2.1		8.3		
Lymphocyte ^c^										
<1000/µL	71	18 (25.4)	0.45	4.9	0.23	2.1	0.13	7.6	0.01	0.07/1.56 (0.97,251)
≥1000/µL	61	20 (32.8)		23		3.6		15.5		
Transplant										
Prior ASCT	40	17 (42.5)	0.04	8.4	0.93	4.6	0.17	10.9	0.18	
Transplant ineligible	94	22 (23.4)		9.7		2.1		7.8		
No. Prior Therapies										
2	79	22 (27.8)	0.85	10.4	0.40	3.7	0.35	9.1	0.76	
≥3	55	17 (30.9)		8.4		2.1		8.2		
Refractory Status ^d^										
Primary	55	12 (21.8)	0.11	10.4	0.75	1.9	0.02	6.6	0.46	
Non-primary refractory	62	23 (37.1)		4.9		3.8		11.1		
Ann Arbor Stage										
1 or 2	33	10 (30.3)	1.00	NR	0.003	4.0	0.04	9.8	0.91	
3 or 4	101	29 (28.7)		4.9		2.3		9.0		
LDH ^e^										
≤ULN	62	26 (41.9)	0.004	10.4	0.98	3.8	0.004	20.8	<0.001	<0.001/2.35 (1.45,3.79)
>ULN	69	12 (17.4)		9.7		1.9		5.4		

Abbreviations: ASCT = autologous stem cell transplant, CrCl = creatinine clearance, DOR = duration of response, LDH = lactate dehydrogenase, No. = number, NR = Not reached, ORR = overall response rate, OS = overall survival, PFS = progression-free survival, ULN = upper limit of normal. ^a^ Comparison was calculated using 2-sided *p*-value (chi-squared test). ^b^ Comparison was calculated using 2-sided *p*-value (log-rank test). ^c^ Data from 2 patients missing. ^d^ Primary refractory is defined as disease progression within 6 months of first-line therapy. Non-primary refractory disease is defined as disease progression ≥6 months after first-line therapy. ^e^ Data from 3 patients missing. Definitions: Overall response rate is the proportion of patients who achieve a partial response or complete response.

**Table 3:** Adverse events (≥10%) based on age and transplant status.

Adverse Event	Age (Years)		Transplant		Total
	<65	≥65	Prior ASCT	Transplant Ineligible	(N = 134)
	*n* (%)		*n* (%)		*n* (%)
Patients with ≥1 AE	51 (98.1)	81 (98.8)	40 (100.0)	92 (97.9)	132 (98.5)
Thrombocytopenia	36 (69.2)	46 (56.1)	35 (87.5)	47 (50.0)	82 (61.2)
Nausea	28 (53.8)	48 (58.5)	25 (62.5)	51 (54.3)	76 (56.7)
Fatigue	24 (46.2)	39 (47.6)	22 (55.0)	41 (43.6)	63 (47.0)
Anaemia	23 (44.2)	34 (41.5)	19 (47.5)	38 (40.4)	57 (42.5)
Decreased appetite	18 (34.6)	31 (37.8)	15 (37.5)	34 (36.2)	49 (36.6)
Diarrhoea	15 (28.8)	31 (37.8)	19 (47.5)	27 (28.7)	46 (34.3)
Neutropenia	18 (34.6)	24 (29.3)	16 (40.0)	26 (27.7)	42 (31.3)
Constipation	17 (32.7)	23 (28.0)	16 (40.0)	24 (25.5)	40 (29.9)
Weight decreased	13 (25.0)	27 (32.9)	12 (30.0)	28 (29.8)	40 (29.9)
Vomiting	11 (21.2)	27 (32.9)	11 (27.5)	27 (28.7)	38 (28.4)
Pyrexia	12 (23.1)	17 (20.7)	9 (22.5)	20 (21.3)	29 (21.6)
Asthenia	8 (15.4)	20 (24.4)	8 (20.0)	20 (21.3)	28 (20.9)
Cough	10 (19.2)	14 (17.1)	8 (20.0)	16 (17.0)	24 (17.9)
Dizziness	7 (13.5)	12 (14.6)	5 (12.5)	14 (14.9)	19 (14.2)
Upper respiratory tract infection	10 (19.2)	9 (11.0)	6 (15.0)	13 (13.8)	19 (14.2)
Hypotension	7 (13.5)	10 (12.2)	7 (17.5)	10 (10.6)	17 (12.7)
Oedema peripheral	3 (5.8)	13 (15.9)	5 (12.5)	11 (11.7)	16 (11.9)
Hyponatraemia	5 (9.6)	10 (12.2)	2 (5.0)	13 (13.8)	15 (11.2)
Dyspnoea	5 (9.6)	9 (11.0)	3 (7.5)	11 (11.7)	14 (10.4)

Abbreviations: AE = adverse event, ASCT = autologous stem cell transplant.

**Table 4:** Summary of selinexor reduction, interruption, and duration.

Variable	No. of Patients	Dose Reduction *n* (%)	Dose Interruption/Withheld *n* (%)	Duration of Selinexor Treatment Median Weeks (min, max)
Overall	134	65 (48.5%)	86 (64.2%)	9 (1, 193)
Age				
<65 years	52	26 (50.0%)	31 (59.6%)	13.5 (1, 193)
≥65 years	82	39 (47.6%)	55 (67.1%)	8 (1, 124)
CrCl				
≤60 mL/min	37	17 (45.9)	25 (67.6)	8 (1, 124)
>60 mL/min	97	48 (49.5)	61 (62.9)	9 (1, 193)
DLBCL Subtype				
GCB	63	28 (44.4)	40 (63.5)	10 (1, 193)
non-GCB	66	33 (50.0)	43 (65.2)	8 (1, 183)
Lymphocyte				
<1000/µL	71	239 (40.8)	50 (70.4)	9 (1, 193)
≥1000/µL	61	34 (55.7)	34 (55.7)	9 (1, 183)
Transplant				
Prior ASCT	40	26 (65.0)	30 (75.0)	16 (1, 183)
Transplant ineligible	94	39 (41.5)	56 (59.6)	9 (1, 193)
No. Prior Therapies				
2	79	38 (48.1)	53 (67.1)	9 (1, 193)
≥3	55	27 (49.1)	33 (60.0)	8 (2, 183)
Refractory Status ^a^				
Primary refractory	55	25 (45.5)	35 (63.6)	9 (1, 183)
Non-primary refractory	62	32 (51.6)	45 (72.6)	11 (1, 124)
Ann Arbor Stage				
1 or 2	33	20 (60.6)	22 (66.7)	9 (1, 183)
3 or 4	101	45 (44.6)	64 (63.4)	9 (1, 193)
LDH ^b^				
≤ULN	62	37 (59.7)	45 (72.6)	15 (1, 193)
>ULN	69	27 (39.1)	41 (59.4)	6 (1, 95)

Abbreviations: ASCT = autologous stem cell transplant, CrCl = creatinine clearance, GCB = germinal center B-cell; LDH = lactate dehydrogenase, max = maximum, min = minimum, No. = number, ULN = upper limit of normal. ^a^ Primary refractory is defined as disease progression within 6 months of first-line therapy. Non-primary refractory disease is defined as disease progression ≥6 months after first-line therapy. ^b^ Data missing for 3 patients.

## Data Availability

The data presented in this study are available on request.
